# Editorial: Biomimetic and bioinspired membranes to reconstruct the properties of natural systems

**DOI:** 10.3389/fmolb.2022.1079570

**Published:** 2022-11-16

**Authors:** Víctor G. Almendro Vedia, Fernando Martínez-Pedrero, Armando Maestro, Eduardo Guzmán, Paolo Natale

**Affiliations:** ^1^ Departamento de Farmacia Galénica y Tecnología Alimentaria, Universidad Complutense de Madrid, Madrid, Spain; ^2^ Departamento de Química Física, Facultad de Ciencias Químicas, Universidad Complutense de Madrid, Madrid, Spain; ^3^ Centro de Fisica de Materiales (CSIC, UPV/EHU)-Materials Physics Center MPC, San Sebastián, Spain; ^4^ IKERBASQUE—Basque Foundation for Science, Bilbao, Spain; ^5^ Unidad de Materia Condensada, Instituto Pluridisciplinar, Universidad Complutense de Madrid, Madrid, Spain

**Keywords:** integral membrane proteins, biomimetic model membranes, nanomaterials, nanoparticles, protein dynamics, membrane dynamics, sphingolipid, cardiolipin

Biological membranes are selectively permeable membranes that separate the interior of a cell from the external environment or adjacent cells. They are micrometre-sized structures based on two-dimensional assemblies of phospholipids carrying integral and peripheral proteins that play a crucial role for cellular function and are the core of cellular communication through direct contact or transport of chemical compounds or ions. All these phenomena are strongly correlated with the interactions of the membrane with lipids, proteins, cofactors, or ions present inside the cell or the extra-cellular environment (Anson, 2009). To better understand the interactions of protein and lipid interfaces of natural membranes, it is common to study the surfaces of systems obtained upon the reconstruction and reconstitution of membranes derived from microorganisms, organelles, macrophages, stem, sperm, or red blood cells. Knowledge on lipid-lipid or protein-lipid interactions should be optimal for several potential technological exploitations, as artificial membrane-like structures which are advantageous vehicles or containers for drug delivery (Peetla et al., 2009; Demetzos, 2015), providing sufficient and specific contact or interaction sites for physiological functioning, along with a fluid matrix for proteins to rotate or diffuse. Accordingly, colloidal and interfacial models, including lipid monolayers, supported lipid bilayers or (proteo-)liposomes, have emerged as indispensable tools to assess physico-chemical properties during fundamental membrane associated processes such as tethering, adhesion, or fusion of bio-membranes (Clifton et al., 2020). The modular nature of membranes allows for the surface engineering of the lipid or protein components, and recently, the replacement of protein or lipid components by the incorporation of nanomaterials, such as block polymers, has become a topic of debate (Bates and Bates, 2017) as they alter specific physiological parameters of the membrane such as endurance, thickness, lipid ordering, or lateral pressure; all very important, mouldable and innate features of an active and floppy bio-membrane. In the field of drug delivery, it has been shown that this can improve some parameters of the lipid membrane, such as the *in vivo* stability and the residence time during circulation through a viscoelastic medium, i.e., body fluids such as blood plasma, interstitial fluid or lymphatic fluid (Grassiri et al., 2021). Membrane engineering might also allow to overcome the limitation imposed by the scallop theorem, which states that a micro-object exhibiting time-symmetric motion cannot achieve net displacement in a viscous environment (Qiu et al., 2014). This Research Topic illustrates the current state-of-the-art and confirms the robustness of some techniques used to measure the properties of biomimetic membranes (further denoted as mimetic membranes or mimetic systems to avoid confusion with bio-membrane). As mentioned above, understanding the kinetics and dynamics of strong or transient membrane interactions requires direct measurement of lipid-lipid, protein-protein or lipid-protein interactions in native environments (Dam et al.), as well as to assess the spatial distributions and lateral diffusion of proteins and lipids present in natural membranes. In this regard, the use of supported lipid bilayers (SLB) in combination with atomic force microscopy, quartz crystal microbalance and fluorescence microscopy has proven to be a powerful tool (Bonet et al.). On the other hand, the use of tailored small, large or giant unilamellar lipid vesicles (SUVs, LUVs or GUVs, respectively), also often referred to as liposomes, constituted a good starting point to carry out studies on the activation energies needed to trigger membrane fusion events, including membrane reshaping and promotion of membrane pore opening.

The use of molecular simulations that introduce possible hydrophobic defects of the lipid membrane bilayer allows, in combination with a reconstituted membrane model, to measure nucleation kinetics that boost lipid reorganization through a synergistic process (François-Martin et al.). Biometric models based on the construction of supported bilayers, or the formation of GUVs have been shown to constitute one of the most powerful models to achieve a large set of mechanical parameters of a system. Their size (1–100 μm) almost allows to reach the scale of a natural membrane system and permits direct visualization and manipulation. Hence, it is possible to obtain in a straightforward and reliable way mechanical parameters such as bending modulus or membrane viscosity. Nonetheless, the interpretation of the physico-chemical data also depends on the quality of the extracted bio-membrane. The extraction and purification of natural membranes is addressed by the separation and isolation of the inner and outer mitochondrial membranes (IMMs and OMMs, respectively) (Schiaffarino et al.). The paper demonstrates that a basic, but rigorous characterization of the native bio-membrane through conventional preparative procedures, that focus on lipid, protein or sugar content, is key to ensure a correct interpretation of the biophysical parameters obtained from the bio-membrane. Using this protocol as a general guideline for the extraction of other native bio-membranes, allows the extraction and initial biophysical study of the material of interest without the need to reconstruct them bottom-up by mixing their individual components. The normalization of this type of approaches will contribute to the expansion of research on natural membranes in combination with mimetic systems.

In summary, the study of the biological membranes using mimetic models requires broad perspective, which is currently structured as a multidisciplinary, including both theoretical and experimental approaches to provide a suitable solution to very complex problems. The study on natural membranes goes hand-in-hand with the development of successful mimetic artificial membranes. Here recent research focuses on some aspects such as membrane plasticity to protect a liposome cargo, the control and targeting at will of the liposomes, or their biocompatibility, which can be improved by mimicking intrinsic characteristics of the surface exposed proteins and lipids. This Research Topic focuses on providing a comprehensive perspective of the current understanding on the study of mimetic membranes, contributing to bridge the gap between the most fundamental aspects of these model systems, and their potential implications in understanding the biophysical aspects involving bio-membranes.
